# Problematic trading: gambling-like behavior in day trading and cryptocurrency investing

**DOI:** 10.47626/2237-6089-2023-0623

**Published:** 2024-11-06

**Authors:** Thiago Henrique Roza, Hermano Tavares, Felix Henrique Paim Kessler, Ives Cavalcante Passos

**Affiliations:** 1 Universidade Federal do Paraná Departamento de Psiquiatria Curitiba PR Brazil Departamento de Psiquiatria, Universidade Federal do Paraná (UFPR), Curitiba, PR, Brazil.; 2 Hospital de Clínicas de Porto Alegre Centro de Pesquisa Clínica Centro de Pesquisa Experimental Porto Alegre RS Brazil Laboratório de Psiquiatria Molecular, Centro de Pesquisa Experimental (CPE) and Centro de Pesquisa Clínica (CPC), Hospital de Clínicas de Porto Alegre (HCPA), Porto Alegre, RS, Brazil.; 3 Universidade Federal do Rio Grande do Sul Departamento de Psiquiatria Porto Alegre RS Brazil Programa de Pós-Graduação em Psiquiatria e Ciências do Comportamento, Departamento de Psiquiatria, Universidade Federal do Rio Grande do Sul (UFRGS), Porto Alegre, RS, Brazil.; 4 Universidade de São Paulo Departamento de Psiquiatria São Paulo SP Brazil Departamento de Psiquiatria, Universidade de São Paulo (USP), São Paulo, SP, Brazil.; 5 USP Instituto de Psiquiatria São Paulo SP Brazil Programa de Dependência Comportamental e Programa de Distúrbios do Controle de Impulsos, Instituto de Psiquiatria, USP, São Paulo, SP, Brazil.

In general terms, financial investments can be understood as the acquisition of an asset, with the aim of generating income from the increase in the asset's value over time, also known as appreciation.^1^ In this context, investing is an essential activity for generation of wealth, with several potential benefits for society at large, although it may be linked to inequalities in some cases. Investments allow companies to grow and start new ventures (by using investors’ money, acquired by selling stocks) at the same time that investors can profit from their investments in those companies.^2^ Over the past few years, there has been significant growth in the number of individual investors.^3^ One of the factors associated with this phenomenon is the internet, with the increasing amount of social media content aimed at new investors as well as user friendly and accessible trading platforms that can even be utilized with a smartphone.^4–6^

Several types of investments, mainly long-term forms that are mostly influenced by knowledge and skill, present a number of positive characteristics such as sizable returns, low risk, and high economic utility.^7^ However, although financial investment and gambling are essentially different activities, there is a considerable overlap between them, mainly when it comes to financial speculation (which involves higher risk and shorter term investments), such as day trading and cryptocurrency investing, and both can be associated with social and mental health burden.^8,9^

## Day trading

Day trading is a type of investment activity (widely considered to be speculation) characterized by rapid financial movements in which stocks are bought and sold on the same day, with the potential for gaining and losing larger amounts of money in short periods of time.^8,10,11^ This model of investing is usually not sustainable over the long term even though it can yield some initial non-negligible gains, which is why some people encourage this activity as a way to make a living.^12^ Nevertheless, there is evidence that higher frequency of stock trading is associated with worse financial returns, with day trading commonly being considered one of the highest frequency types of trading, in which only a minority of day traders achieve profits over time.^2^ For instance, according to empirical data from Brazil, 97% of investors (who began trading on the Brazilian market between 2013 and 2015) who persisted in this type of investment for more than 300 days ended up losing money, with the probability of an individual profiting from this type of investment decreasing with the number of days they persisted trading.^12^

Day trading presents similarities with some types of gambling, mainly with online and skill-based gambling.^10^ Even though day trading is not solely based on chance, due to its characteristic of short time between purchases and sales, it is often vulnerable to sudden price changes.^13^ In this sense, it is similar to sports betting (which is often reliant on the study of sports statistics before bets), in which there is a "technical analysis" prior to the act of investing in a specific stock. Even though they may be helpful in some cases, these analyses often do not anticipate sudden market changes, which can lead to significant monetary losses.^13^ In addition, in the case of some day traders, the main motive for stock market investments may be associated with thrill-seeking and sensation-seeking feelings similar to craving, rather than the desire to make profits.^5,11^

A study from Australia (n = 9,245 adults) reported that day traders were predominantly male, over the age of 45 years, lived with a partner, and had high income and high educational attainment.^8^ In addition, more than 90% of the day traders in their sample reported that they also participated in gambling activities, with the prevalence of problem gambling among day traders being 7.6%, with both proportions being significantly higher in comparison with non-day traders.^8^ The same study also described that day traders showed a preference for skill-based types of gambling.^8^

According to a case series of excessive traders (n = 8, all males), problematic trading shares many characteristics with gambling disorder, such as psychiatric comorbidities, impulsivity, trajectories, and diagnostic criteria; these patients also experience small wins early on, chase losses, exhibit poor assessment of risks and an illusion of control over outcomes, and lose control over their money.^11^ These patients also focused on riskier investments, which allow for higher gains, but are also vulnerable to higher losses.^11^ In addition, these patients spent considerable amounts of time daily on trading activities (neglecting other areas of life), and highly valued social status and success, being highly competitive.^11^ Therefore, reported negative consequences were not rare and varied from marital conflicts, financial losses, and lawsuits to adverse psychological outcomes.^11^

Finally, from the stock market perspective, day trading is strongly associated with other speculative and risky behaviors, such as preference for assets on the options market with returns distributions resembling lottery betting that could lead to instability in stock prices.^14^

## Cryptocurrency investing

In general terms, cryptocurrency represents a relatively new technology in finance, being defined as a decentralized digital currency based on technologies such as cryptography and blockchain, allowing for financial transactions between two parties in anonymity and without the need for intermediary financial institutions.^15–17^ Bitcoin, invented in 2008 by Satoshi Nakamoto (a pseudonym representing a person or group of people who signed the paper "Bitcoin: A Peer-to-Peer Electronic Cash System") is the best known cryptocurrency, but is only one of the thousands of different digital coins that exist.^16,17^ The cryptocurrency industry is growing rapidly with huge increases in market capitalization over the past decade.^16^ Several reputable companies and major banks have started accepting cryptocurrency as a valid method for payments and financial transactions; in addition, there has recently been a discussion about the role of cryptocurrency payments in healthcare economics, ranging from payment for outpatient appointments to surgical and other medical procedures.^15,18,19^

Cryptocurrencies are generally considered to be highly volatile assets, with the potential to generate high returns in short periods of time, as well as high losses, which is the reason why they are widely considered to be risky and speculative investments.^20–23^ There is a considerable difference between cryptocurrency investing and stock trading. For instance, cryptocurrency trading is more often an individual activity, by people who invest in preliminary concepts and narratives, which can in some cases even be memes, in contrast to those who invest in well-established and solid companies.^21^ In addition, cryptocurrency trading is more influenced by social media activity in comparison with other types of investments.^21^

Taking this context into account, a parallel can be drawn between cryptocurrency investing and gambling-like behaviors in the sense that both rely on the expectation of uncertain, but high gains.^21^ Additionally, over the past few years, empirical research has shown evidence of an association between cryptocurrency investing and gambling.^21,24^ For instance, a preliminary cross-sectional investigation based on an online sample of regular gamblers (n = 876) reported that cryptocurrency investing was a common practice among participants (prevalence of approximately 53%) and that cryptocurrency trading was significantly and positively associated with problem gambling severity.^24^ Also, the same study reported a marked overlap between cryptocurrency investing and high-risk stock trading among regular gamblers.^24^ Another empirical study (n = 543 participants) also described a significant association between higher problem gambling scores and intensity of cryptocurrency trading.^9^ Moreover, according to a recent scoping review, there is an association between problematic gambling and intensity or involvement in cryptocurrency trading, which can be seen as a different expression of gambling in several cases.^23^ For instance, individuals who engage in cryptocurrency trading are less prone to long-term investments, often report higher financial losses, exhibit "loss-chasing" and "money-borrowing" behaviors, and present similarities with gamblers in terms of personality (impulsivity and novelty-seeking) and some sociodemographic characteristics (predominance in the male sex and in younger age groups).^23^ In addition, the same study reported that individuals who engage in cryptocurrency trading report significantly higher psychological distress and loneliness, even in comparison with individuals who engage in other types of financial investments.^23^

Several risk factors have been associated with problematic cryptocurrency trading, including some that constitute similarities with online gambling.^13^ Illusion of control (manifesting as strategies, rituals, or specific skills, which may give an individual investor the illusion of higher returns), social learning and reinforcement (mainly due to social media content and influencers), fear of missing out (regretting not having made a specific investment or sale in a specific time period), and chasing one's losses are examples of these potential risk factors.^13^

Another point to consider regarding cryptocurrency is its association with criminal activity. Cryptocurrency frauds and scams, ranging from phishing, Ponzi schemes (in which very high profits are promised for investors, with payments for older investments being paid with money received by new investors) and pyramid schemes, to market manipulation, have been growing in frequency and magnitude over the past years, inflicting considerable financial losses on victims, and becoming an issue of significant concern for governments and the private sector.^13,16^

## Clinical implications and future prospects

Even though there is not yet enough evidence to support a distinct diagnosis of problematic trading, it is important to assess these patients comprehensively. For those investigating patients with gambling-like trading behavior, one option is to adapt the Diagnostic and Statistical Manual of Mental Disorders, 5th edition, Text Revision (DSM-5-TR) criteria for gambling disorder to the patients’ context.^11,25^ In addition, it is essential to map these problematic trading behaviors (the potential harm of these behaviors can vary substantially depending on several factors such as type of investment, amount invested, and short-term vs. long-term investments, among others), and investigate their consequences in physical (including daily habits) and mental health terms, as well as their impact on financial, occupational, family, and social life.^23^ Another essential aspect is to investigate depressive symptoms and suicide risk in these patients, mainly after suffering significant losses.^26^ The act of constantly checking price movements, which may happen even in the case of casual investors, is another problematic behavior that may impact one's sleep cycle, productivity, and overall functionality.^13^

There is preliminary evidence that patients with problematic trading rarely seek treatment, which could be associated with lack of insight or stigma towards mental health problems, which may compromise their status and career.^5^ In addition, retail traders (individual traders) are usually more vulnerable to developing problematic trading behavior and losing money in comparison with institutional traders.^11^ Nevertheless, it is essential to differentiate those who exhibit gambling-like behaviors in financial investments from those who have lost significant amounts of money for other reasons while trading, since the latter may not necessarily represent a problematic pattern of trading, even though these patients may face significant distress and may meet criteria for other psychiatric conditions (anxiety, depression and suicidality, for instance) due to their losses.^27^

Another essential point is the need for more research in this field. For instance, there is a discussion about the role of modern trading apps, such as Robinhood, which are similar to gambling apps and may promote problematic trading behavior in individual users.^5^ Moreover, day trading and cryptocurrency related content is very common on social media platforms (for instance, YouTube and Reddit) and may be responsible for stimulating more riskier investments practices, in some cases even being associated with investments in highly volatile "meme assets" such as the Dogecoin cryptocurrency and GameStop stocks, with several members of these social media communities (such as the WallStreetBets subreddit) calling their investing behavior gambling.^4,23^ Regulations, harm prevention, financial literacy, and public health policies targeting the population vulnerable to this type of behavior should also be investigated in future studies.^4,5^

There is a need to investigate these emerging phenomena in Brazil and other countries in Latin America. For instance, according to data from B3 S.A. (Brasil, Bolsa, Balcão, the Brazilian stock exchange), the number of individual investors increased approximately 300% from 2018 (814 thousand investors) to 2020 (3.2 million investors) and the number is still rising.^3^ Even though more women have started investing in recent years, most individual investors in Brazil are male (approximately 74%) and mean age is 32 years.^6^ In addition, the majority of these Brazilian investors learned about investments from social media influencers and on YouTube channels.^6^ Finally, there was an increase in interest and prevalence of trading practices during the coronavirus disease 2019 (COVID-19) pandemic, which may ultimately have contributed to the increase in problematic trading.^10^ Nevertheless, there is a need for robust data to enable more definitive conclusions to be drawn about the connection between the COVID-19 pandemic and problematic trading behaviors in order to develop prevention strategies and interventions for this vulnerable population.
